# Prognostic nutritional index predicts clinical outcome in patients with acute ST-segment elevation myocardial infarction undergoing primary percutaneous coronary intervention

**DOI:** 10.1038/s41598-017-03364-x

**Published:** 2017-06-12

**Authors:** Qing-Jie Chen, Hui-Juan Qu, Dong-Ze Li, Xiao-Mei Li, Jia-Jun Zhu, Yang Xiang, Lei Li, Yi-Tong Ma, Yi-Ning Yang

**Affiliations:** 1 Department of Cardiology, First Affiliated Hospital of Xinjiang Medical University, Urumqi, China; 2 Xinjiang Key Laboratory of Cardiovascular Disease Research, Urumqi, China; 3 Department of Cardiology, West China Hospital, Sichuan University, Chengdu, China

## Abstract

We aimed to investigate whether the prognostic nutritional index (PNI), a combined nutritional-inflammatory score based on serum albumin levels and lymphocyte count, was associated with mortality in patients with acute ST-segment elevation myocardial infarction (STEMI) undergoing primary percutaneous coronary intervention (pPCI). From September 2011 to November 2014, 309 consecutive patients with STEMI undergoing pPCI were prospectively enrolled. Patients with a combined score of albumin (g/L) + 5 × total lymphocyte count × 10^9^/L ≥ 45 or <45 were assigned a PNI score of 0 or 1, respectively. Of the 309 STEMI patients, 24 (7.74%) died in the hospital, and 15 (4.83%) died during long-term follow-up (median follow-up time, 19.5 [3–36] months). Compared to patients with a PNI of 0, patients with a PNI of 1 had significantly higher in-hospital (14.2% vs. 3.7%; *P* < 0.001) and long-term follow-up (21.7% vs. 6.9%, *P* < 0.001) mortality rates. PNI (1/0, HR, 2.414; 95% CI, 1.016 to 5.736; *P* = 0.046) was a significant independent predictor of mortality in patients with STEMI undergoing pPCI. Moreover, cumulative survival was significantly lower for patients with a PNI of 1 compared to patients with a PNI of 0 (78.3% vs. 93.1%, log-rank *P* < 0.001). PNI appears useful for the risk stratification of STEMI patients undergoing pPCI.

## Introduction

Despite recent advances in techniques and improved outcomes of percutaneous coronary intervention (PCI), patients with acute ST-segment elevation myocardial infarction (STEMI) are still at an increased risk of mortality even after timely revascularization^1–4^. Therefore, early risk stratification at the time of presentation is of clinical importance.

Malnutrition has been recognized as an independent risk factor for morbidity and mortality in patients with chronic heart failure (CHF)^5, 6^. Some single nutritional indices such as albumin have been shown to be closely associated with poor outcomes in patients with CHF^7, 8^. Additionally, to evaluate nutritional status, more complex indices, such as the prognostic nutritional index (PNI), have been developed and widely used^9–13^. The PNI is a combined nutritional-inflammatory score based on serum albumin levels and the lymphocyte count that reflects the immunological nutritional condition and that measures the risk of several types of cancer in patients^14^. This index is convenient to obtain since only simple blood biomarkers are required.

The prognostic influence of nutritional status in cardiovascular disease (CVD) is not very well understood. It seems that lower serum albumin levels are increased risk factors for coronary disease, which could, together with traditional risk factors, assist in confirming patients who are at risk of myocardial infarction (MI)^8, 9^. However, one study has shown that hypoalbuminemia was related to adverse events but not to mortality in patients with acute coronary syndrome. The impact of hypoalbuminemia on outcomes after STEMI has not been comprehensively investigated. Older patients may be especially vulnerable to haemodynamic changes, and we speculated that hypoalbuminemia and PNI may have markedly adverse effects on older patients with STEMI. Therefore, the aim of the present study was to examine whether the PNI on admission was associated with mortality in patients with acute STEMI undergoing pPCI.

## Results

Between September of 2011 and November of 2014, we conducted a follow-up study at the Department of Cardiology, the First Affiliated Hospital of Xinjiang Medical University. A total of 324 consecutive patients who presented with acute STEMI within 12 hours of undergoing pPCI at our institution were enrolled. Among these patients, 15 patients were excluded from the final analysis according to our exclusion criteria. Thus, 309 patients were included in the final analysis; of these, 189 (61.2%) patients had a PNI of 0, and 120 (38.8%) patients had a PNI of 1on admission.

Patient baseline clinical characteristics by PNI score are shown in Table 1. Compared to patients with a PNI of 0, those with a PNI of 1 had an advanced age (*P* = 0.034), more often had a Killip class >2 (*P* = 0.004), had a higher GRACE score (*P* = 0.037), and had a lower LVEF (*P* = 0.002). Patient baseline biochemical characteristics by PNI score are shown in Table 2. Patients with a PNI of 1 had significantly lower blood albumin (*P* < 0.001), lower triglycerides (*P* < 0.001), lower total cholesterol (*P* = 0.014), lower haemoglobin levels (*P* < 0.001), and lower WBC counts (*P* = 0.025) compared with those with a PNI of 0.

**Table 1:** Baseline clinical characteristics of patients with acute STEMI after pPCI by PNI score group.

Variable	PNI = 0 (n = 189)	PNI = 1 (n = 120)	χ2/t	P
Age, years	56 ± 12	62 ± 11	−4.10	0.034
Males, n (%)	162 (85.7)	88 (73.3)	0.01	6.504
Diabetes mellitus, n (%)	46 (24.3)	31 (25.8)	0.03	0.872
Hypertension, n (%)	102 (54.0)	51 (42.5)	3.86	0.049
Smoking, n (%)	119 (63.0)	64 (53.3)	3.46	0.178
Admission SBP, mmHg	125 ± 19	120 ± 18	2.35	0.020
Admission DBP, mmHg	77 ± 14	73 ± 12	2.18	0.030
Admission heart rate, beats/min	79 ± 14	78 ± 13	0.69	0.490
Killip class (class ≥2)	23 (12.2)	28 (23.3)	13.16	0.004
In-hospital medications				
Aspirin, n (%)	189 (100)	118 (98.3)	1.11	0.292
β-blockers, n (%)	180 (95.2)	115 (95.8)	0.06	0.806
ARB/ACEI, n (%)	182 (96.3)	112 (93.3)	0.83	0.363
CCB, n (%)	83 (43.9)	45 (37.5)	0.99	0.319
Tirofiban, n (%)	184 (97.4)	117 (97.5)	0.006	0.937
Medications at discharge				
Aspirin	182 (96.3)	116 (96.7)	0.03	0.864
Clopidogrel	188 (99.5)	119 (99.2)	0.34	0.561
ACEI/ARB	155 (82.0)	98 (81.7)	0.01	0.939
β-blockers	170 (89.9)	104 (86.6)	4.56	0.033
LVEF, %	58.39 ± 6.29	55.95 ± 7.36	3.11	0.002
Coronary artery disease, n (%)				
Left main	13 (6.9)	5 (4.2)	0.55	0.458
Left anterior descending	146 (77.2)	88 (73.3)	0.42	0.518
Left circumflex	100 (52.9)	61 (50.8)	0.06	0.811
Right coronary artery	113 (59.8)	76 (63.3)	0.25	0.615
Gensini score	57.16 ± 34.50	58.51 ± 36.42	0.76	0.307
GRACE score	143 ± 25	149 ± 24	2.09	0.037

SBP, systolic blood pressure; DBP, diastolic blood pressure; ARB, angiotensin II receptor blocker; ACEI, angiotensin-converting enzyme inhibitor; CCB, calcium-channel blocker; GRACE, the Global Registry of Acute Coronary Events; IABP, intra-aortic balloon pump counterpulsation; LVEF, left ventricular ejection fraction; PNI, prognostic nutritional index; STEMI, acute ST-segment elevation myocardial infarction.

**Table 2:** Laboratory findings in STEMI patients after pPCI by PNI score group.

Variable	PNI = 0 (n = 189)	PNI = 1 (n = 120)	χ2/t	P
WBC, ×109/L	11.3 ± 3.64	10.3 ± 3.63	2.26	0.025
Lymphocyte count, ×109/L	2.34 ± 1.25	1.30 ± 0.57	8.58	<0.001
Neutrophil count, ×109/L	8.03 ± 3.45	8.38 ± 3.75	−0.84	0.40
Mean platelet volume, ×1012/fL	10.3 ± 1.2	10.6 ± 1.5	−2.02	0.045
RDW, %	4.8 ± 0.56	4.4 ± 0.62	−2.29	0.023
Haemoglobin, g/L	147 ± 17	133 ± 19	6.71	<0.001
Creatinine, μmol/L	83.4 ± 42.09	85.5 ± 52.05	−0.38	0.701
Blood urea nitrogen, mmol/L	5.2 ± 1.55	5.1 ± 2.33	0.36	0.722
Triglycerides, mmol/L	2.3 ± 1.78	1.4 ± 0.87	5.50	<0.001
Total cholesterol, mmol/L	4.6 ± 1.01	4.3 ± 1.13	2.47	0.014
Low-density lipoprotein cholesterol, mmol/L	2.8 ± 0.80	2.8 ± 0.81	0.33	0.742
Troponin T, ng/mL	0.21 ± 0.66	0.23 ± 1.31	−0.62	0.535
CK, IU/L	251 (99–910)	418 (98–1495)	−0.99	0.319
CK-MB, U/L	28 (17–98)	44 (19–144)	−1.19	0.233
Serum albumin, g/dL	40.8 ± 5.39	34.3 ± 4.66	10.10	<0.001
Urine protein positive, (%)	33 (17.5)	33 (27.5)	4.40	0.036

WBC, white blood cell; RDW, red blood cell distribution; CK, creatinine kinase; CK-MB, creatinine kinase – myocardial band isoenzyme; PNI, prognostic nutritional index; STEMI, acute ST-segment elevation myocardial infarction.

**Figure 1:**
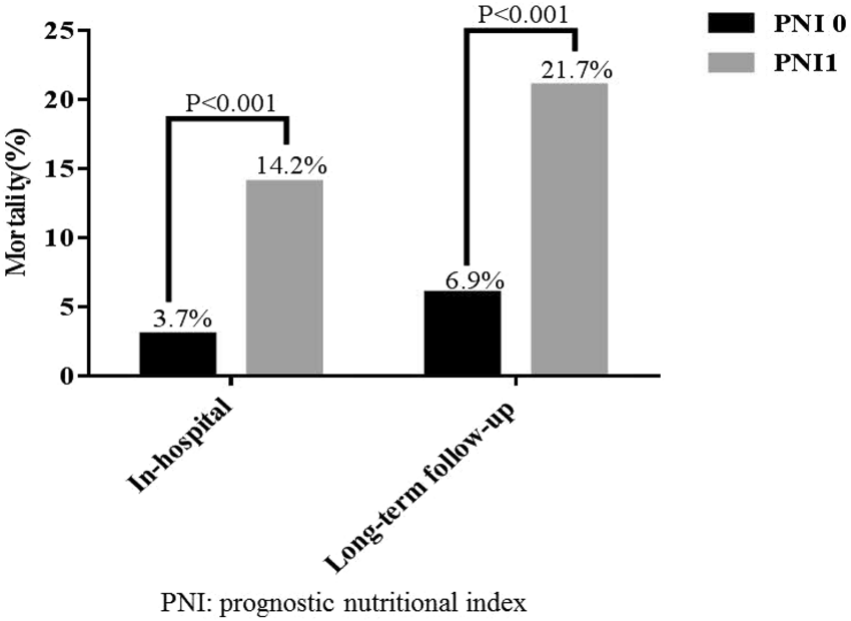
In-hospital and long-term follow-up mortality in the PNI = 0 and PNI = 1 groups of STEMI patients who underwent pPCI.

Compared to PNI = 0 group, the PNI = 1 group had significantly higher in-hospital mortality (14.2% vs. 3.7%, *P* < 0.001; Fig. 1). Consistently, the PNI = 1 group showed significantly higher mortality (21.7% vs. 6.9%, *P* < 0.001) compared with the PNI = 0 group at long-term follow-up (median follow-up time, 19.5 [3–36] months).

**Figure 2:**
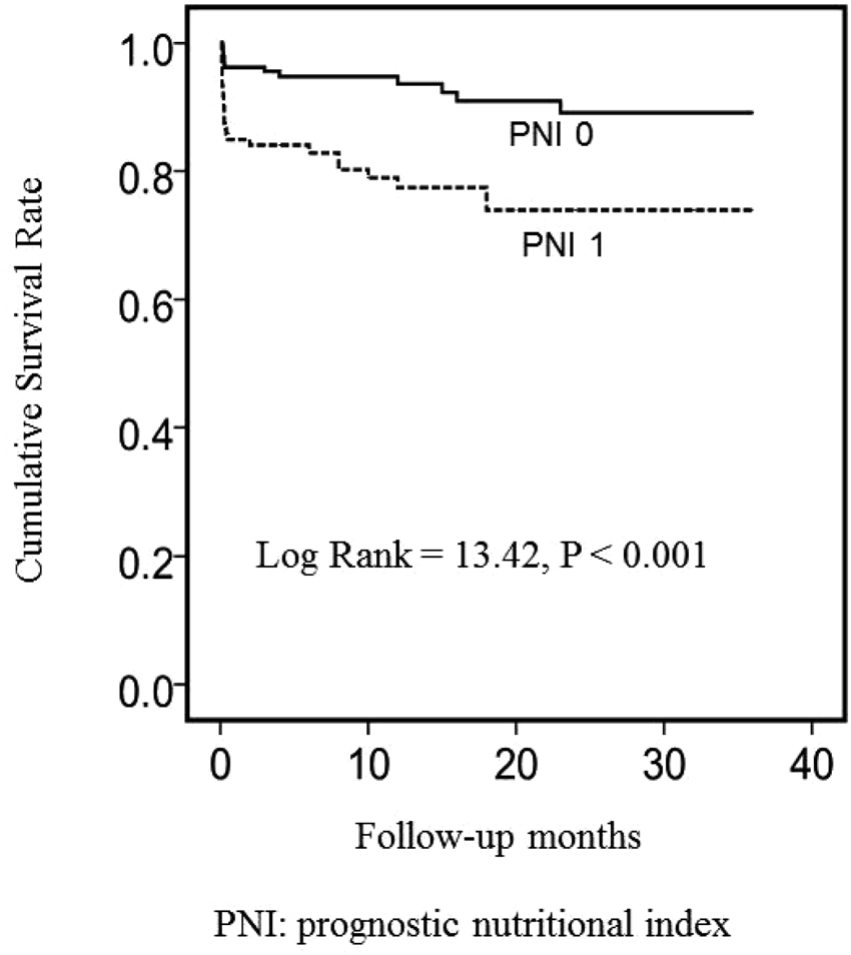
Kaplan–Meier survival analysis using the PNI score group of the STEMI patients.

In univariate and multivariate analyses (Table 3), a greater PNI (1 vs. 0) on admission was a significant predictor of mortality (hazard ratio, 3.232 [95% confidence interval, 1.661–6.292], *P* = 0.001; and 2.414 [1.016–5.736], *P* = 0.046, respectively). Other multivariate independent predictors of mortality were age (hazard ratio, 1.064 [95% confidence interval, 1.027–1.102], *P* = 0.001), LVEF (hazard ratio, 0.931 [95% confidence interval, 0.890–0.974], *P* = 0.002), WBC count (hazard ratio, 1.203 [95% confidence interval, 1.118–1.294], *P* < 0.001) and GRACE score (hazard ratio, 1.038 [95% confidence interval, 1.002–1.074], *P* < 0.001). Moreover, cumulative survival was significantly lower in patients with a PNI of 1 relative to patients with a PNI of 0 (78.3% vs. 93.1%, respectively; log-rank *P* < 0.001) (Fig. 2).

**Table 3:** Independent predictors of mortality in the Cox proportional hazard model.

Variable	Univariate analysis			Multivariate analysis		
	HR	95% CI	P	HR	95% CI	P
Age	1.065	1.033–1.097	<0.001	1.064	1.027–1.102	0.001
AST	1.002	1.000–1.004	0.020	1.002	0.997–1.006	0.408
LDH	1.001	1.000–1.002	0.002	1.000	0.998–1.002	0.953
PNI (1 vs. 0)	3.232	1.661–6.291	0.001	2.414	1.016–5.736	0.046
Red blood count	0.450	0.280–0.721	0.001	0.372	0.101–1.364	0.136
Haemoglobin	0.980	0.966–0.944	0.004	1.028	0.981–1.077	0.242
Blood urea nitrogen	1.215	1.066–1.385	0.003	0.980	0.817–1.175	0.828
Creatinine	1.005	1.002–1.008	<0.001	1.005	0.999–1.012	0.104
Triglycerides	0.690	0.495–0.960	0.028	0.805	0.544–1.193	0.280
TC	0.708	0.509–0.986	0.041	0.856	0.569–1.289	0.457
LVEF	0.916	0.883–0.950	<0.001	0.931	0.890–0.974	0.002
Urine protein	1.402	1.064–1.846	0.016	0.914	0.411–2.034	0.826
Left main	1.670	0.593–4.703	0.332	1.908	0.454–8.020	0.378
WBC	1.149	1.070–1.235	<0.001	1.203	1.118–1.294	<0.001
Killip class ≥2	5.15	1.350–18.378	0.016	2.525	0.629–10.130	0.191
Gensini score	1.03	0.989–1.020	0.603	1.001	0.984–1.017	0.963
GRACE	1.014	1.011–1.037	<0.001	1.038	1.002–1.074	<0.001

AST, aspartate transaminase; LDH, lactate dehydrogenase; TC, total cholesterol; LVEF, left ventricular ejection fraction; WBC, white blood cell count; GRACE, Global Registry of Acute Coronary Events; PNI, prognostic nutritional index; HR, hazard ratio; CI, confidence interval.

As shown in Table 4, the PNI = 1 group had significantly higher rates of in-hospital all-cause mortality (14.2 vs. 3.7%) (*P* < 0.001 for all), of bleeding (6.7 vs. 3.7%), and of recurrent MI (5.0 vs. 2.1%); however, there was no significant difference between the two groups in terms of the rates of reinfarction or bleeding. At long-term follow-up (median follow-up time, 19.5 [3–36] months), the PNI = 1 group showed significantly higher rates of mortality (7.5 vs. 3.2%, *P* < 0.001) and had a lower frequency of cerebrovascular accidents (0.8 vs. 1.1%, *P* < 0.001), whereas there was no difference with respect to myocardial infarction re-interventionon percutaneous coronary intervention.

**Table 4:** Major adverse cardiac events in STEMI patients after pPCI by PNI score group.

	PNI = 0 (n = 189)	PNI = 1 (n = 120)	P-value
In-hospital complications			
Bleeding	7 (3.7)	8 (6.7)	0.363
In-hospital mortality	7 (3.7)	17 (14.2)	<0.001
Recurrent MI	4 (2.1)	6 (5.0)	0.286
MACE (follow-up)			
MI	8 (4.2)	7 (5.8)	0.714
Death	6 (3.2)	9 (7.5)	<0.001
CVA	2 (1.1)	1 (0.8)	<0.001
Re-intervention PCI	11 (5.8)	6 (4.6)	0.958

CVA, cerebrovascular accident; MACE, major adverse cardiac events; MI, myocardial infarction; PCI, percutaneous coronary intervention; PNI, prognostic nutritional index; STEMI, acute ST-segment elevation myocardial infarction.

## Discussion

To the best of our knowledge, this is the first study to investigate the prognostic value of PNI, a combined nutritional-inflammatory score based on serum albumin levels and the lymphocyte count, in patients with STEMI undergoing pPCI. A greater PNI of 1 on admission was associated with higher in-hospital and long-term follow-up mortality in STEMI patients treated with pPCI. PNI was identified as a significant independent predictor of mortality in STEMI patients undergoing pPCI. Moreover, cumulative survival was significantly lower in patients with a PNI of 1 relative to patients with a PNI of 0. Thus, PNI on admission appears to be useful for risk stratification of STEMI patients undergoing pPCI.

The PNI combines the albumin level and the lymphocyte count into a single composite marker of nutrition, inflammation and immunity status^10, 15^. The PNI was initially designed to assess the immunological and nutritional aspects of patients who underwent gastrointestinal tract surgery and was predominantly an indicator of the nutritional status of any given patient^16–18^. Albumin is a widely used indicator for nutrition and has been shown to correlate with postoperative complications^19, 20^. However, there is increasing evidence that the serum albumin level decreases with the increasing severity of inflammation^9, 21, 22^. Albumin not only is the main determinant of plasma oncotic pressure but also transports numerous substances and participates in both acute and chronic inflammatory responses^23, 24^. Many studies have shown an elevation of inflammatory markers in myocardial infarction, and several inflammatory markers have been identified as useful predictors of clinical outcomes^25, 26^. Meanwhile, there is increasing evidence that hypoalbuminemia on admission is a strong independent predictor of long-term mortality and severe heart failure development in pPCI treated STEMI patients^9^. Moreover, hypoalbuminemia was significantly associated with in-hospital mortality in patients with STEMI who were ≥85 years of age^27^.

Atherosclerotic plaque rupture is an inflammatory process that is mediated by the complex interaction between neutrophil-mediated reactive immune responses and subsequent lymphocyte-mediated adaptive immune responses^28–30^. Procoagulants are secreted locally by the leukocytes, increasing oxidative and proteolytic damage^31, 32^. Lymphocytes also have an essential role in modulating the inflammatory response at different stages of the atherosclerotic process^28^. In the acute setting of coronary events, lymphocytopenia is a common finding during the stress response and is secondary to increased corticosteroid levels, whereas a greater lymphocyte count reflects a more appropriate immune response and a stable, quiescent inflammatory status^33^. In two previous studies, lymphocytopenia was independently associated with the occurrence of complications and death after acute myocardial infarction^34, 35^.

In our study, we found that PNI was an independent predictor of mortality in patients with STEMI undergoing pPCI. However, the pathophysiological mechanism of the association between the higher PNI and the poorer clinical outcome of STEMI patients who are treated with pPCI are not completely understood. Because PNI is calculated based on the serum albumin level and the total lymphocyte count in the peripheral blood, PNI may characterize both the nutritional and immunological status, which could affect the survival rate of cancer patients^15, 36, 37^. Several mechanisms may be responsible. First, increased inflammatory activity in the setting of STEMI may be the underlying mechanism for the association between a decreased serum albumin (SA) level and mortality after pPCI^38^. Additionally, SA is an important inhibitor of platelet aggregation that increases the production of the antiaggregatory prostaglandin D2 (PGD2) from cyclic endoperoxides^39, 40^. Furthermore, hypoalbuminemia may increase blood viscosity and disrupt endothelial function due to the increased concentrations of free lysophosphatidylcholine^41^. In addition, the lymphocyte count is an index of cell-mediated immunity. A low lymphocyte count may be associated with pre-existing immunosuppression, which could indicate an inadequate immunological reaction in the patient^42^. Therefore, in theory, combining serum albumin levels and the lymphocyte count to create the PNI may be able to estimate the nutritional, inflammatory and immunity statuses of STEMI patients. In the present study, the area under the ROC curve for the prediction of mortality using PNI exceeded the area under the ROC curve for the prediction of mortality using albumin levels and the lymphocyte counts separately, which indicates that the PNI provides a stronger predictive power of mortality than do its components.

The present study has several limitations. First, it is a single-centre study, and findings in the present study need to be confirmed and validated in larger populations. Second, inflammation-associated markers other than WBC counts were not analysed or compared with the PNI. Third, only baseline serum albumin levels and baseline lymphocyte counts were determined, and serial measurements of the latter PNI components may provide an additional prognostic value. Finally, future prospective studies are warranted to clarify the pathophysiologic roles of the PNI components.

Our study indicated that the PNI is a significant prognostic factor for STEMI patients undergoing pPCI. Proper assessments of the nutritional-inflammatory status of STEMI patients at the time of hospital admission can help to minimize (if not prevent) complications and readmission and mortality rates. The proof that the aggressive treatment of malnutrition, in addition to personalized dyslipidaemic therapy, in STEMI patients improves outcomes would be the most convincing evidence. Therefore, more comprehensive studies on the treatment of malnutrition and its effect on outcomes are needed.

## Methods

### Study design

This prospective cohort study complied with the Helsinki Declaration of 1975 based on its 1983 revision, and the Human Ethics Committee of the First Affiliated Hospital of Xinjiang Medical University approved the study protocol. All patients provided written informed consent. The study inclusion criteria were as follows: presentation within the first 12 hours of the onset of chest pain and ST-segment elevation of at least 1 mm in two or more contiguous leads (2 mm for leads V_1_–V_3_) or presentation with a new-onset left bundle branch block. Patients with an active infection, a previous chronic inflammatory disease history, a known malignancy, an advanced-stage liver or renal disorder, or fibrinolytic administration in the previous 30 days were excluded from this study. PNI was assessed as a predictor of in-hospital and long-term follow-up mortality as the primary outcome.

### Study subjects

From September 2011 to November 2014, a total of 324 consecutive patients who were hospitalized at the First Affiliated Hospital of Xinjiang Medical University for acute STEMI and who underwent pPCI within 12 hours of the diagnosis were screened for study enrolment. Of these patients, 15 patients were excluded: 3 patients had unavailable laboratory data, 10 patients were treated with urgent coronary artery bypass graft surgery due to failed pPCI or due to coronary anatomy that was not amenable to pPCI, 1 patient had severe renal failure, and 1 patient had a chronic infection. Consequently, a total of 309 patients were enrolled in this study.

### Data collection and blood sampling

The baseline clinical and demographic characteristics of the patients were recorded from hospital files and computer records. Venous blood samples were collected using standardized EDTA blood tubes within 1 hour of admission. Albumin levels were measured using the photometric method (DC 800; Beckman Coulter, Dublin, Ireland). A haematology profile including the lymphocyte count, haemoglobin level and mean platelet volume (MPV) was determined using an automated haematology analysis system (LH750, Beckman Coulter Inc., Brea, CA, USA). An electrocardiogram (ECG) was immediately obtained on admission, 60 minutes after the procedure, and daily thereafter during the length of the hospital stay. Left ventricular ejection fraction (LVEF) was calculated using the biplane Simpson method within 12 hours of admission.

### Coronary angiography and percutaneous coronary intervention

All patients received a 300 mg dose of chewable aspirin and a 300–600 mg loading dose of clopidogrel on admission. Prior to the procedure, patients also received 70 U/kg of standard intravenous heparin. The use of a glycoprotein IIb/IIIa inhibitor (tirofiban) was left to the discretion of the primary operator. All pPCI procedures were performed by experienced interventional cardiologists who used a transradial approach and drug-eluting stents. Serial monitoring of cardiac biomarkers was performed every eight hours starting 24 hours after the PCI and continuing until the patient was discharged. After the intervention, all patients received daily regimens of 100 mg of aspirin and 75 mg of clopidogrel. At the time of discharge, all patients were prescribed a daily aspirin regimen in perpetuity and a daily regimen of clopidogrel for at least 9–12 months.

### Long-term follow-up

In-hospital clinical data were recorded from hospital files and computer records. The long-term follow-up (median: 19.5 months) data of patients were obtained from follow-up visits or in-hospital clinical records of re-admitted patients. In addition, all patients were contacted by telephone.

### Definition of PNI

Baseline total lymphocyte counts and albumin levels were routinely measured on admission. The PNI cut-off score of 45 has been used in 3 recent oncological studies and has been confirmed as optimal^15, 27, 33^. Based on those results, we choose a score of 45 as our tangent point value. The PNI was calculated as described previously: albumin (g/L) + 5 × total lymphocyte count × 10^9^/L scores that were ≥45 and scores that were <45 at admission were assigned PNI scores of 0 (n = 189) and 1 (n = 120), respectively.

### Statistical analysis

Continuous variables are expressed as either the mean ± SD or the median with an interquartile range (25–75%). Parametric, non-parametric and categorical data were compared using one-way ANOVA, Kruskal-Wallis, and chi-square (χ^2^) tests, respectively. Kaplan-Meier curves were generated for each PNI group. Cox proportional hazard models were used to investigate whether PNI predicted mortality during the study period. Forty variables were tested as all-cause mortality predictors in the univariate analysis, and the 14 significant variables were included in the multivariate analysis. *P* < 0.05 was considered statistically significant. Data analysis was performed using the SPSS version 18.0 statistical software package (Chicago, IL, USA).
